# Supplementary figures and images for: Characterising plasmacytoid and myeloid AXL+ SIGLEC-6+ dendritic cell functions and their interactions with HIV
Source: PLoS Pathog. 2024 Jun 26;20(6):e1012351. doi: 10.1371/journal.ppat.1012351 (PMC11233022; doi:10.1371/journal.ppat.1012351)

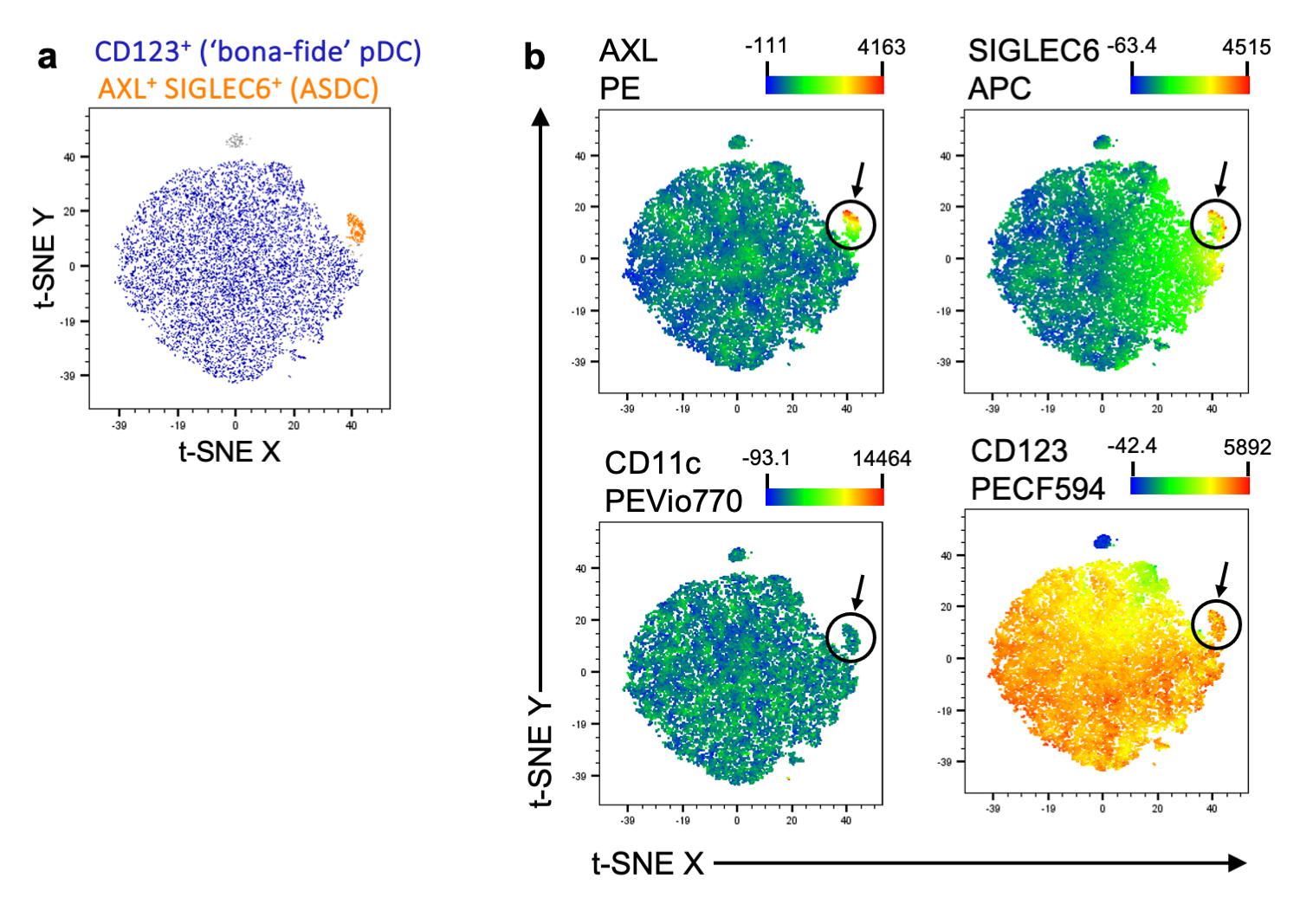

Supplement: S1 Fig — t-stochastic distributed neighbour embedding (t-SNE) analysis performed on live single Lin1-HLA-DR+ cells based on AXL, Siglec-6, CD123, and CD11c. (a) Representative t-SNE dot plot shows the distribution of AXL+ Siglec-6+ DCs (orange) and AXL- Siglec-6- CD123+ pDCs (blue) on t-SNE plot. (b) Heat map visualisations of the median fluorescence intensity of surface AXL, Siglec-6, CD123, and CD11c expression for populations on t-SNE plot. (TIF) [file ppat.1012351.s001.tif]

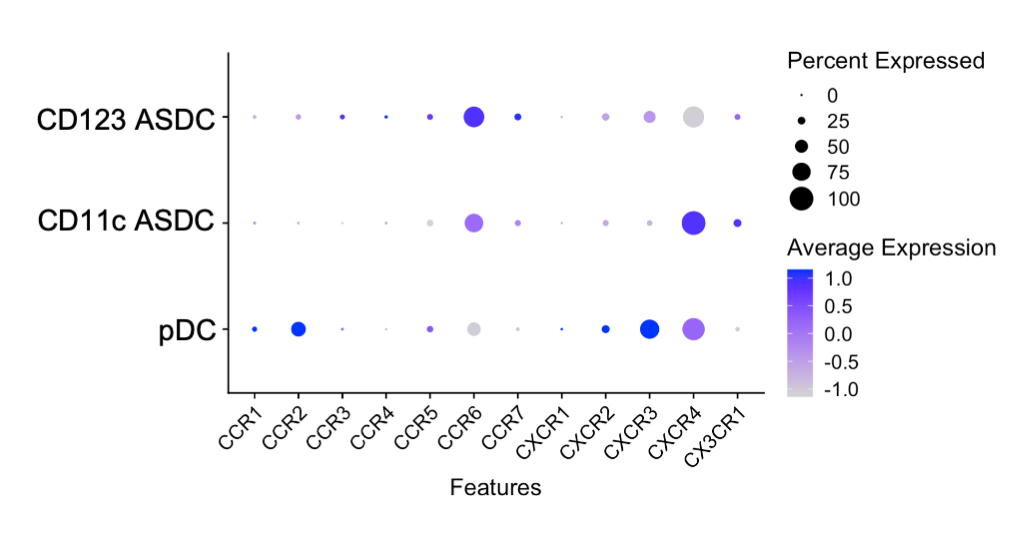

Supplement: S2 Fig — Sorted blood PBMCs were transcriptionally profiled by scRNAseq (GSE94820)[4]. pDC, CD11c+ ASDC and CD123+ ASDC annotations were determined using metadata provided by the authors. Chemokine receptors gene expression is shown in pDCs, CD123+ and CD11c+ ASDCs. (TIF) [file ppat.1012351.s002.tif]

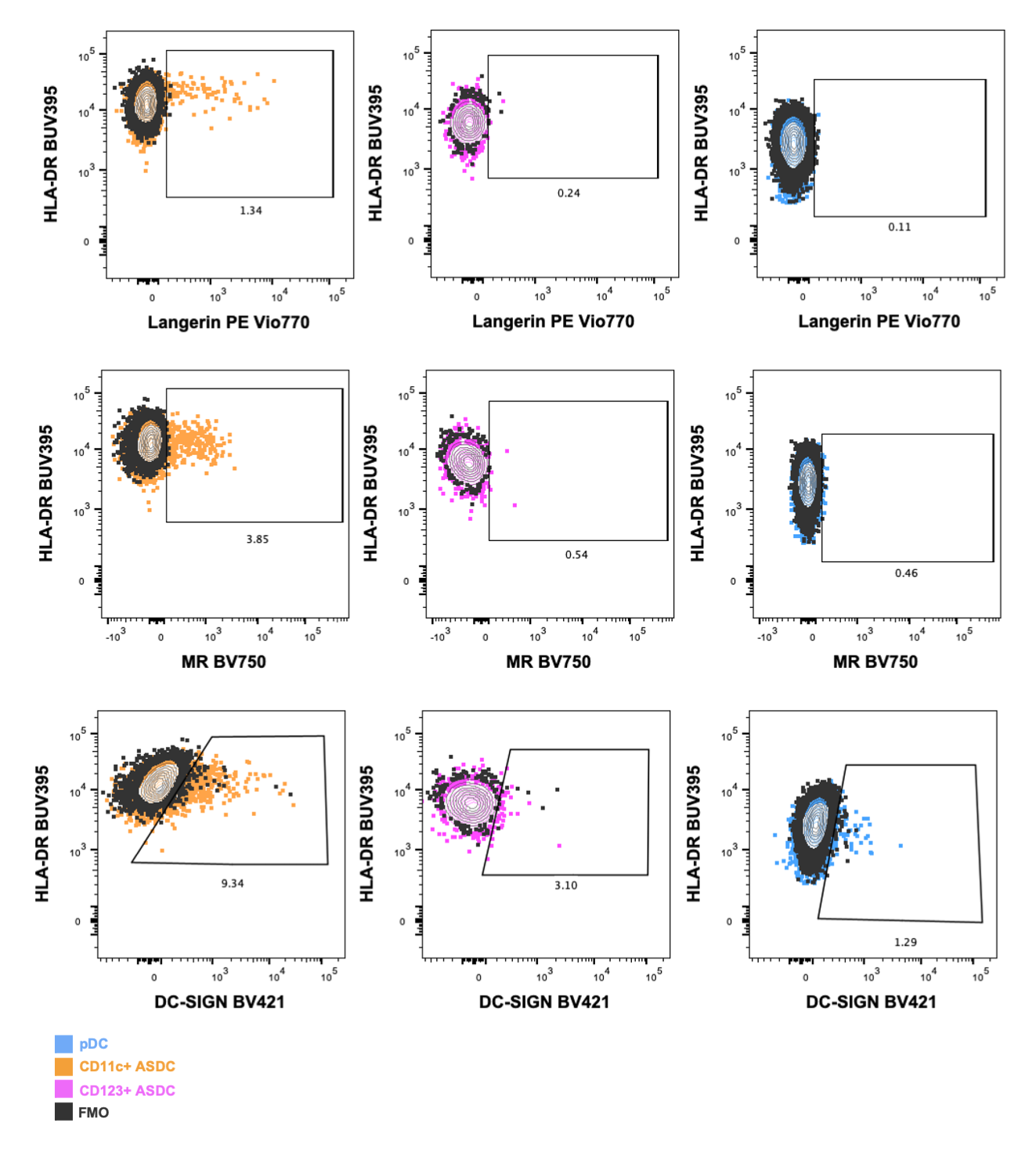

Supplement: S3 Fig — Pan DCs were FACS sorted into CD11c+ ASDCs, CD123+ ASDCs and pDCs and surface stained for flow cytometry. Representative plots show the percentage expression of Langerin, MR and DC-SIGN displayed as an FMO (grey) overlayed with true expression for each population. As the percent of positive population was small, expression was displayed as a percentage rather than gMFI. (TIF) [file ppat.1012351.s003.tif]

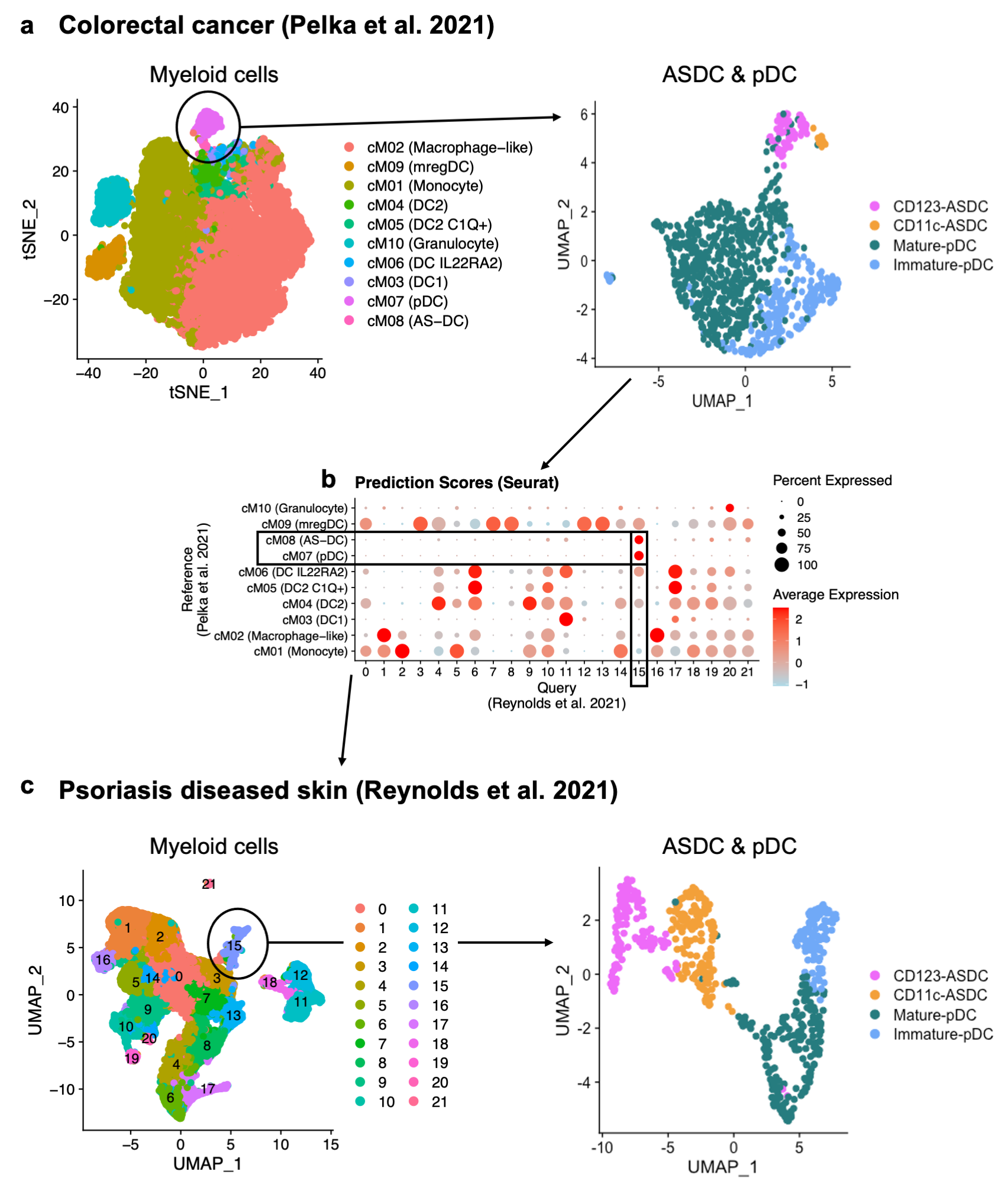

Supplement: S4 Fig — (a) Pelka et al. 2021 data was downloaded from the GEO (GSE178341). Myeloid clusters (cM01-10) were isolated and analysed separately. The tSNE coordinates used by the authors were downloaded from the Broad Institute’s Single Cell Portal (SCP1162). ASDCs and pDCs described by the authors were isolated and reclustered for analysis. (b-c) Reynolds et al. 2021 data was downloaded from Developmental Human Cell Atlas (E-MTAB-8142). Myeloid cells, as annotated by the authors, were isolated and analysed separately. Data was batch corrected using Seurat’s RPCA method, and clustered using a resolution of 0.6, resulting in 22 clusters. Seurat’s FindTransferAnchors and TransferData functions were used to determine predicted annotations, with GSE178341 (or colorectal cancer) acting as the reference data to E-MTAB-8142 (or psoriasis skin) as the query. A DotPlot was used to demonstrate the prediction scores generated by the functions. ASDCs and pDCs, identified as cluster 15, were isolated and reclustered for analysis. (TIF) [file ppat.1012351.s004.tif]

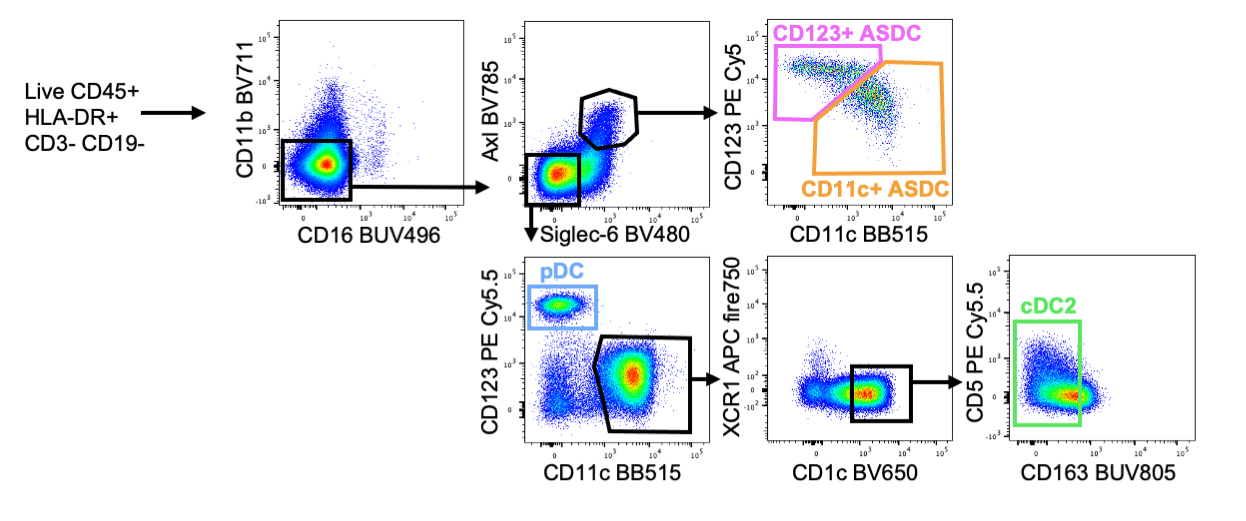

Supplement: S5 Fig — Blood pan DCs were isolated and stained for flow cytometry using a modified panel to allow for the identification of cDC2s. All cells were first gated as Live CD45+ HLA-DR+ CD3- CD19-. Subsequent cell populations were identified as CD123+ ASDCS (CD11b- CD16- AXL+ Siglec-6+ CD123+), CD11c+ ASDCs (CD11b- CD16- AXL+ Siglec-6+ CD11c+), pDCs (CD11b- CD16- AXL- Siglec-6- CD11c- CD123+) and cDC2s (CD11b- CD16- AXL- Siglec-6- CD123- CD11c+ CD1c+ XCR1- CD163-). (TIF) [file ppat.1012351.s005.tif]

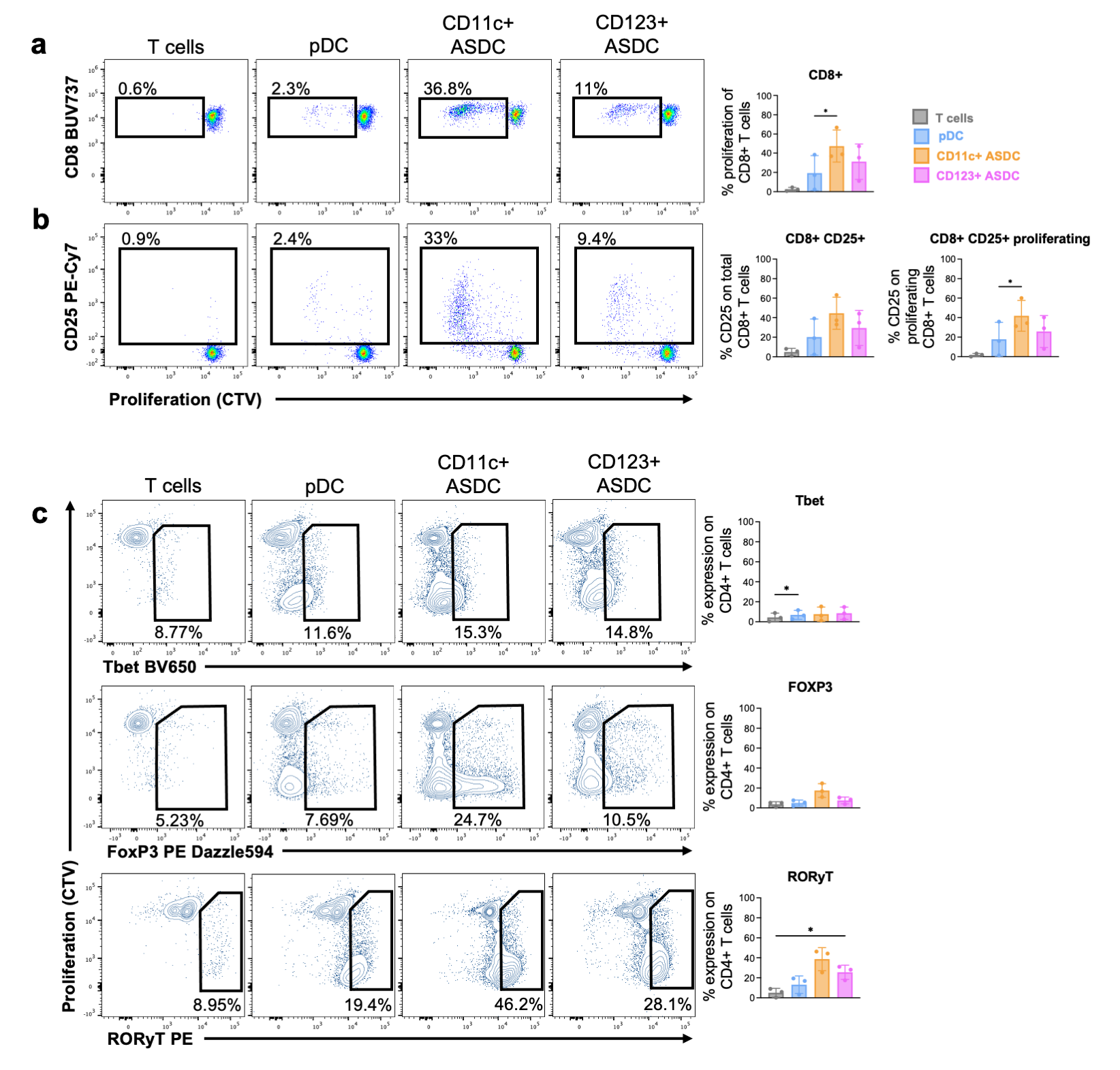

Supplement: S6 Fig — FACS sorted pDCs, CD123+ and CD11c+ ASDC were cultured for 7 days at 37°C with Cell trace Violet-stained naïve T cells at a ratio of 1 ASDC or pDC: 10 T cells. Cultures were analysed by flow cytometry to assess (a) CD8+ T cell proliferation, (b) CD8+ T cell activation via CD25 expression, (c) CD4+ T cells percentage expression of transcription factors Tbet, FoxP3 and RORγT. Data presented as mean of ±SD. For all data, *p < 0.05 using one-way ANOVA with Tukey’s multiple comparisons test. (TIF) [file ppat.1012351.s006.tif]

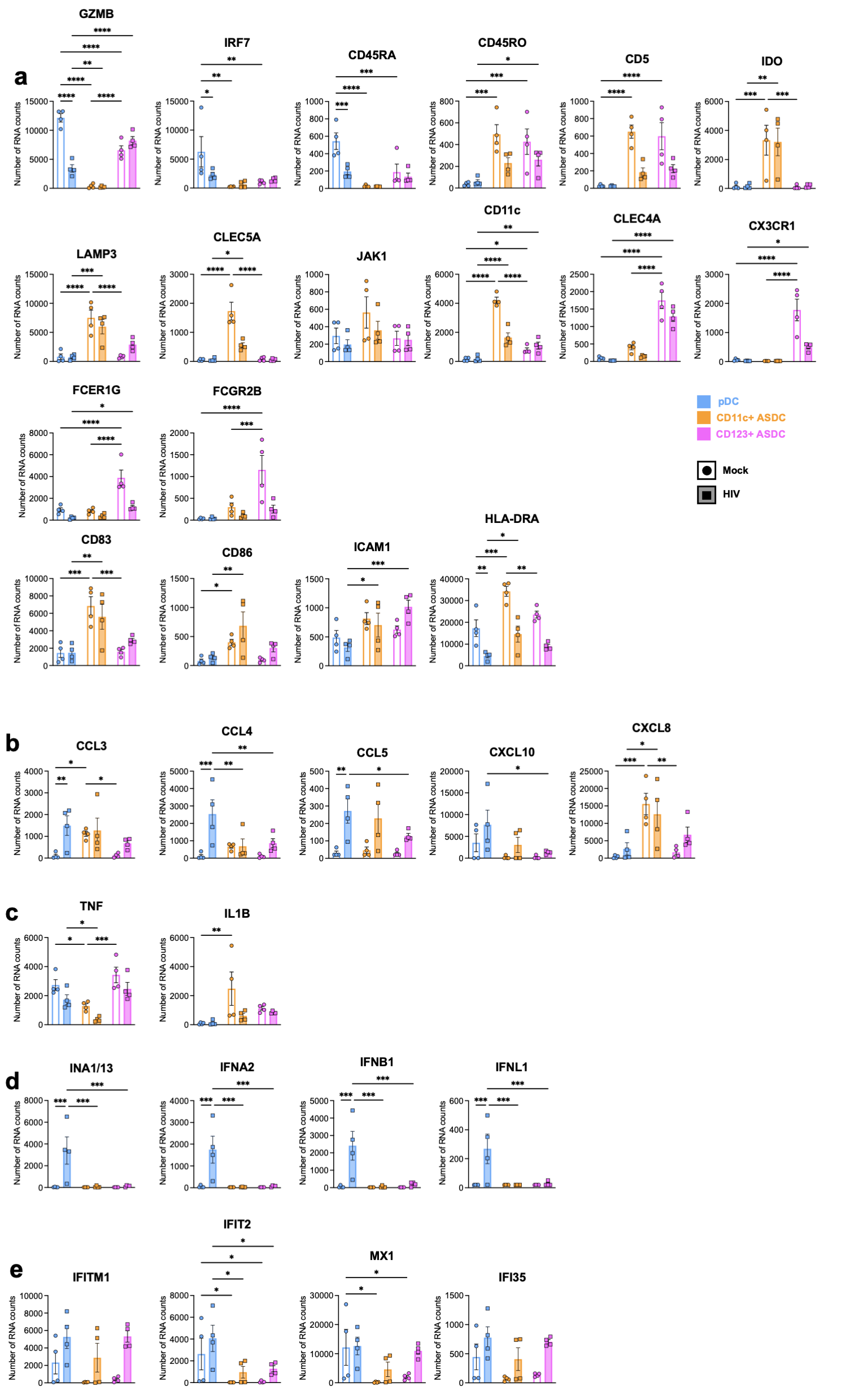

Supplement: S7 Fig — pDCs and ASDCs were profiled using NanoString as either mock or after 18 hours of HIV exposure. (a) Markers defining pDCs, CD11c+ and CD123+ ASDCs; (b) Chemokines (CCL3, 4, 5; CXCL10 and 8), (c) cytokines (TNFα, IL-1B), (d) IFN, (e) ISGs in mock and HIV exposed pDCs and ASDCs. Data presented as mean of ±SD. For all data, *p < 0.05, **p < 0.01, ***p < 0.001, ****p < 0.0001 using one-way ANOVA with Tukey’s multiple comparisons test. (TIF) [file ppat.1012351.s007.tif]

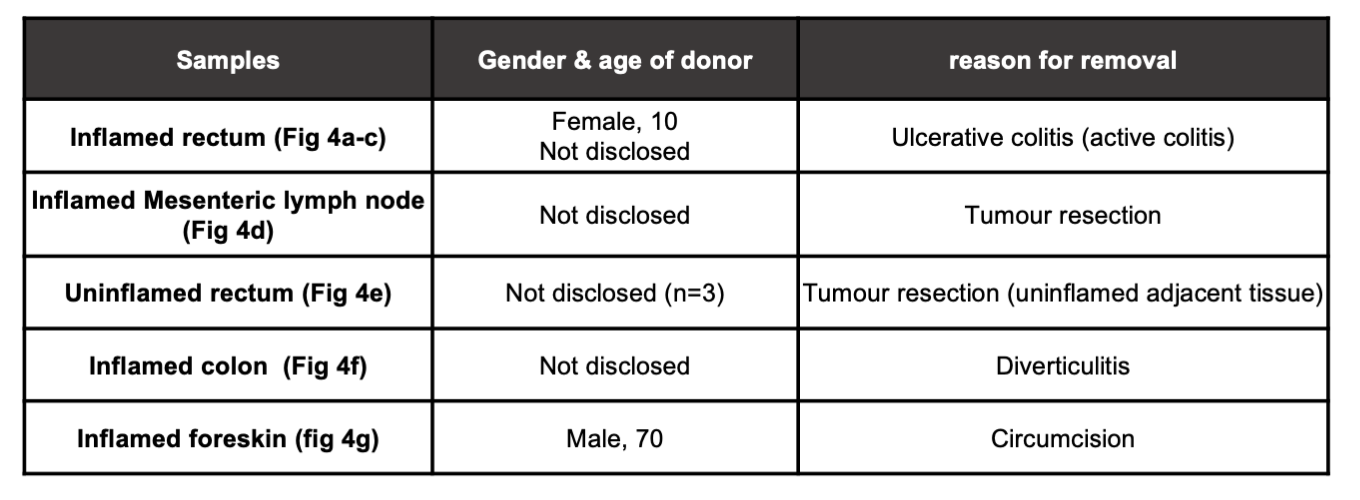

Supplement: S2 Table — Information on tissues collected from patients, their medical condition, age/sex (when disclosed) and reason of tissue removal used in Fig 4a–4g to identify ASDCs and pDCs in inflamed tissues. (TIF) [file ppat.1012351.s009.tif]

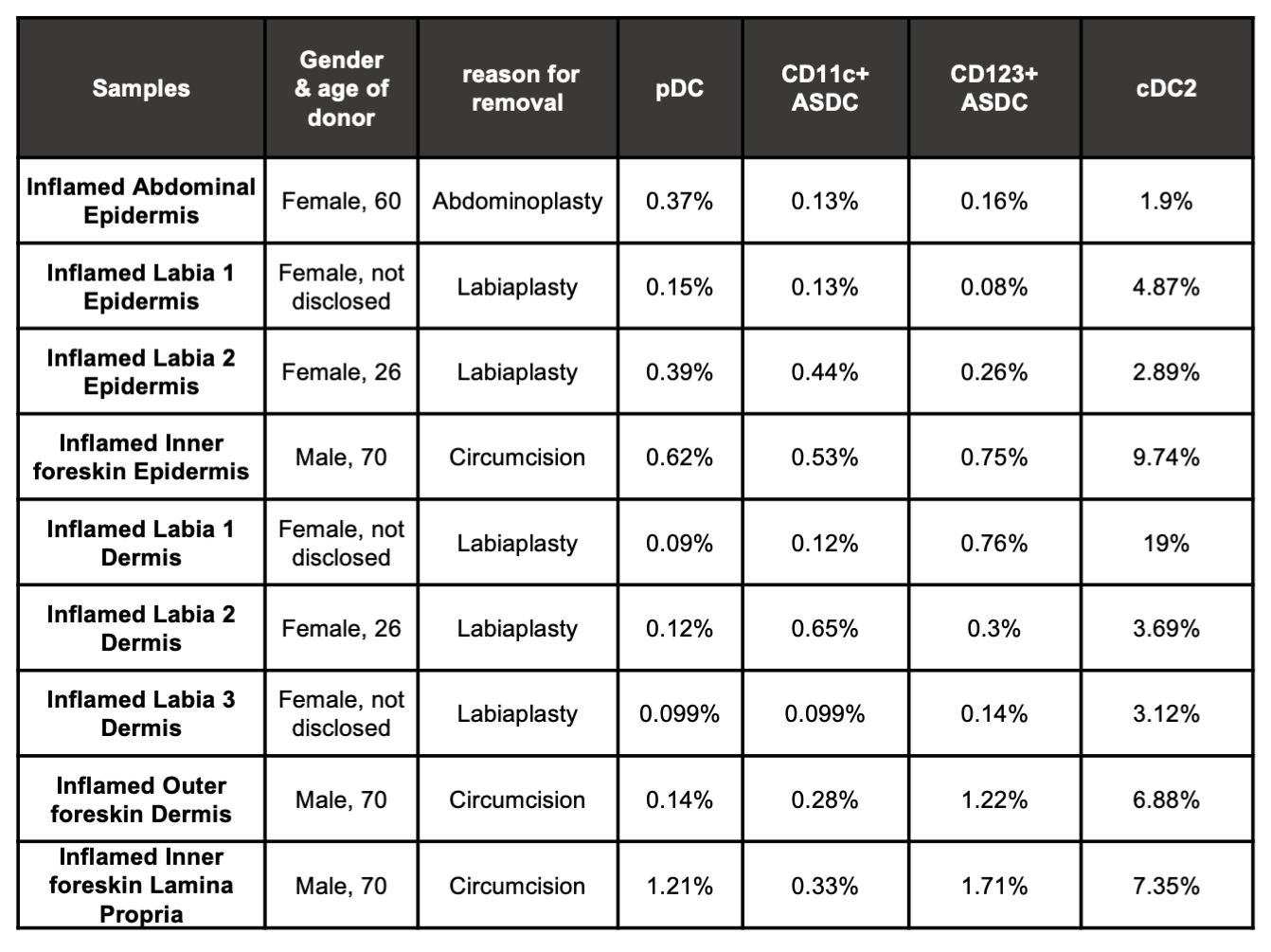

Supplement: S3 Table — Information on tissues collected from patients, their medical condition, age/sex (when disclosed), reason of human genital tissues removal, and the relative proportions of pDCs, ASDCs and cDC2 as a percentage of live CD45+ HLA-DR+ CD3- CD19- cells used in Fig 5 to identify pDCs, ASDCs and cDC2 in inflamed human skin and genital tissues: abdominal epidermis (n = 1), labia epidermis (n = 2), inner foreskin epidermis (n = 1), labia dermis (n = 3), outer foreskin dermis (n = 1) and inner foreskin lamina propria (n = 1). (TIF) [file ppat.1012351.s010.tif]
