# Supplementary material for: Characterising plasmacytoid and myeloid AXL+ SIGLEC-6+ dendritic cell functions and their interactions with HIV
Source: PLoS Pathog. 2024 Jun 26;20(6):e1012351. doi: 10.1371/journal.ppat.1012351 (PMC11233022; doi:10.1371/journal.ppat.1012351)
Supplement: Attachment — Submitted filename: Response to reviewers PLoS Path.pdf [file ppat.1012351.s012.pdf]

Thank you for all the comments. We appreciate all the time and effort the editorial members and reviewers have contributed to the improvement of this manuscript. Below are our responses to each comment in blue font and what have been included in the manuscript is in red font. Line numbers refer to the tracked changes version of the manuscript.

When you are ready to resubmit, please upload the following:
[1] A letter containing a detailed list of your responses to the review comments.
[2] Two versions of the revised manuscript: one with tracked changes denoting where the text has been changed; the other a clean version (uploaded as the manuscript file).

Part I - Summary
Reviewer #1: In this work, the authors have analyzed the Axl+ Siglec6+ dendritic cell subset (AS DC) in human blood and anogenital tissues and assessed their interaction with HIV virus in ex vivo assays. A particular attention was paid to the 2 subpopulations of AS DC (CD11c high or CD123 high).
Main claims are:
1. AS DC are present in inflamed tissues in human
2. AS DC are more efficient than pDC for stimulating T cells and for inducing cytokine polarization
3. Both subpopulations of ASDC can transmit HIV virus using different mechanisms
the novelty of claims 1&2 is compromised by previous publications (which are not properly referenced in the manuscript).

Reviewer #2 Immunophenotyping by flow cytometry was performed in parallel in blood and cells from peripheral tissues obtained from a limited number of donors. some conclusions are not supported by direct evidence, important questions must be addressed to validate the significance of the findings presented and their relevance.

Part II – Major Issues: Key Experiments Required for Acceptance
Reviewer #1: Main issues.
1. The authors claim that AS DC have only been described in blood and tonsils. However, there are several articles showing the presence of bona fide AS DC in spleen (10.1126/science.aag300), bone marrow (10.1126/science.aag300; 10.1016/j.immuni.2017.11.001 (we couldn't find bone marrow in J. immuni), inflamed cerebrospinal fluid (10.1016/j.clim.2023.109686), inflamed broncho-alveolar lavage (10.1038/s41467-019-09913-4), inflamed skin (10.1084/jem.20190811). References need to be updated. We have added all ref to lines 321-322 (except for bone marrow as we couldn't find any ref to it in j. immunity. The claim 'we have shown for the first time that AS DC are present in inflamed human tissues' (line 175) needs to be revised and tuned down). We have deleted for the first time and the sentence now reads (line 434-435) "In summary, we have shown that ASDCs are present in inflamed human anogenital tissues".

2. The analysis shown in figure 3 needs to be better explained.
Why did the authors subset the data on monocytes (line 631 now line 2132 in tracked version)? Thank you for picking up on this. The data was subsetted on myeloid subsets, as described by the authors who annotated the data. We have now changed this to read that the data was subsetted on myeloid cells and made changes to the text to make it clearer (line 2132).
The different sub-setting steps should be shown (at least in supplementary data) to make this analysis more convincing. We have now provided the requested supplementary data as S4 Fig. It shows the original data provided by Pelka et al., and Reynolds et al., and the clusters that represent pDCs and ASDCs that we chose for re-clustering. We have added from line 326 "The sub-setting steps of the single cell RNA sequencing analysis are shown in S4 Fig". In addition, UMAP has now become the

standard representation for this type of analysis instead of t-SNE. tSNE and UMAP are both valid tools for data representation. At the reviewer's request, Figure 3 is now updated to show a UMAP rather than tSNE.

3. The superior ability of AS DC to stimulate T cell proliferation compared to pDC, and the higher ability of CD11c+ AS DC has been shown previously (10.1016/j.immuni.2017.11.001). This needs to be mentioned. We have now acknowledged the similarity of our data to that published by Alcantara-Hernandez et al., by adding on line 576 "aligning with a previous report by Alcantara-Hernandez et al [20].

4. Although the reviewer understands that obtaining human tissues is difficult, it is problematic to draw conclusions from data derived from only one biological sample (figure 8) now figure 5. It is also very difficult to compare this data to blood AS DC and pDC because histograms are not shown in figure 8 (now figure 5). As it stands, the data reported in figure 8 seems rather preliminary. We appreciate the reviewer's acknowledgement of difficulties with tissue availability. We agree this data is preliminary as for most tissue types one biological sample was investigated. However, we do believe that this figure is critical as it demonstrates the presence of both ASDC subsets in a range of human samples and their CLR expression as a proof of principle concept. We have now modified the manuscript to emphasise the preliminary nature of this data in line 444-449: "This preliminary data indicates that blood (Fig 5b) and tissue ASDCs express similar levels of Siglec-1 and CD4. However, expression of the CLRs CD206/MR, CD207/Langerin and CD209/DC-sign trended higher on tissue ASDCs compared to blood. We have also identified cDC2 in tissues (Fig 4g) and blood (S5 Fig). In tissues, cDC2 differed in CLR expression compared to CD11c+ ASDCs (Fig 5) as they had low Siglec-1 and no DC-sign expression."

Reviewer #2

1 The authors assessed the ability of the ASDCs and pDCs to polarize T helper (Th) cells (Figure 5c). Th polarization was evaluated based on the cytokines present in the culture supernatant after a 6-day incubation of T cells with antiCD3/CD28 +/- DC subsets. The authors conclude from these results that Th1 are not induced (line 40, 223, 380). However, Interferon gamma, which is the canonical Th1 cytokine, was not measured. We thank the reviewer for this comment and apologise for this oversight. To detect cytokines, we used the LEGENDplex Hu Th Cytokine Panel that can detect IFN gamma. However, IFN gamma was saturated in the LEGENDplex and was not reported in our submitted manuscript. To overcome saturation, we have now run an IFN gamma ELISA and found high levels of production under all conditions (>15,000 pg/ml). This was added to Fig 6d. To support authors' conclusions, Th polarization should be confirmed by analysing transcription factors and performing intracellular cytokine staining. At the reviewer's request we have now investigated transcription factors added this to the results as S6c Fig. We have also amended the manuscript from line 577 on page 12-13 to read "We also examined CD4 T cell cytokine production and possible subset polarisation by pDCs and ASDCs. pDCs induced low levels of all Th cell cytokines. Compared to T cells that were not polarised with any APCs, ASDCs stimulated low levels of Th1 (TNF-a, IL-2) cytokines and higher levels of Th2 (IL-4, IL-5 and IL-13), Th9 (IL-9) and Th22 (IL-22) cytokines. IL-17 secretion from Th17 and IL-10 from Treg was low but higher than non-polarised T cells. IFN-g secretion was high but similar to non-polarised T cells. Selected expression of transcription factors Tbet, FoxP3, RORγt, which identify Th1, Treg, and Th17, respectively, was examined. ASDCs induced the highest proportion of T cells expressing RORgt followed by FoxP3 and low T bet expression (S6c Fig)."As we have measured the extracellular production of cytokines, we find it redundant to reassess them at the intracellular level.

Throughout the manuscript, data are presented with SEM, which quantifies uncertainty in estimate of the mean. Standard deviation (SD) indicates dispersion of the data from mean (PMID: 23125963). To visualize the variability within sample, descriptive data should be precisely summarized with SD instead of the SEM in Figure 1, 4h, 5-7. We have now replaced SEM by SD in all figs including Supplementary Figs. This has not affected the statistical significance of the results.
The authors utilize the co-expression of Axl and Siglec-6 to define ASDCs similarly to the approach by Villani et al. (PMID: 28428369). Through Tsne representation of their flow cytometry data, they show that ASDCs can be segregated into 2 clusters with differential expression of CD123 and CD11c. Since the authors claim an enrichment of CD11c+ ASDCs in inflamed tissues, it is important to exclude that those cells are distinct from bona fide CD1c+ DC2. As we first started this study and as shown in our gating for figures 1a and 4a-d, no antibodies were included to identify DC2. As our gating strategy in figures 1 (blood) and 4a-e (tissues) resembled that of Figure 6A in PMID28428369, which demonstrates that ASDCs are distinct from DC2, we are confident that CD1c+ DC2, which do not express AXL and SIGLEC6, are not encompassed within our CD11c+ ASDCs in figures 1a and 4a-d.

Figure 6A from PMID 28428369

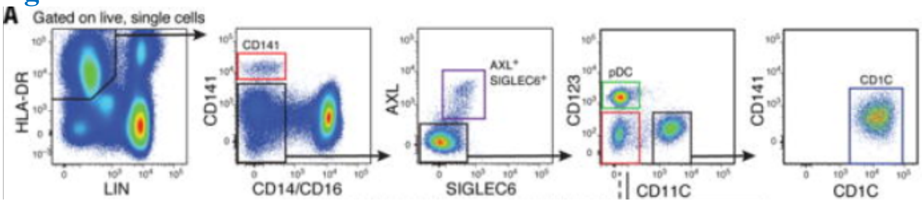

Later in our study, we included markers to identify DC2 in both blood (S5 Fig) and tissues (Fig 4g). In our fig 4g (tissue), we included CD1c in the flow panel and have now amended 4g to accommodate a new gating strategy showing that DC2s are a separate population from CD11c ASDCs. We have now added to line 432-433 on page 11 " cDC2s were additionally identified in these samples as a separate population to ASDCs in much higher numbers". This distinction is crucial to substantiate the authors' conclusions, particularly considering the findings in Figure 2, which indicate that CD11c+ ASDCs express numerous co-stimulatory molecules (lines 135-136), suggestive of activated DC2 cells. Our findings of CD11c+ ASDCs express numerous co-stimulatory molecules is corroborated by data in PMID28428369 Suppl figure 6C showing that ASDCs (referred to as DC5) expressed the costimulatory CD86 like DC2. Furthermore, data in figure 5E from PMID28428369 showed that there is proliferation of allogeneic of T cells 5 days after co-culture with either $CD1C^{+}$ DCs, $CD123^{+}CD11C^{-/lo}$ ASDCs (1, pink bar), or $CD123^{lo}CD11C^{+}$ ASDCs (2, blue bars). Therefore, ASDCs are mature in phenotype similar to DC2.

Suppl figure 6C from PMID2842836 **Figure 5E from PMID 28428369**

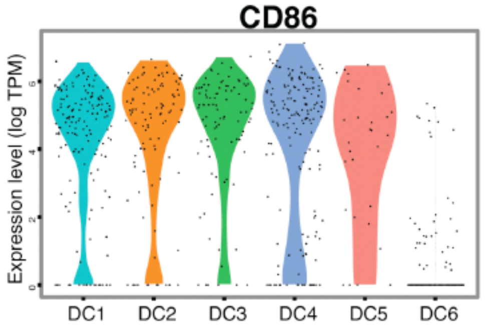

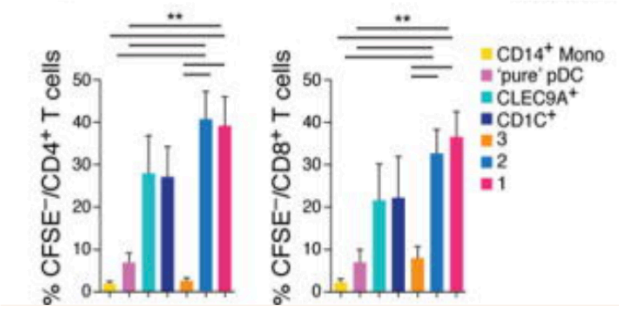

Based on all the above we are confident that our gated CD11c+ASDCs are not activated DC2. This has been now emphasised by adding to the discussion in lines 1050-1051 page 16 "Furthermore, our findings shows that ASDCs are a different population than cDC2 and that they are mature in phenotype like cDC2s" and on page 16 lines 1060-1062 "Thus, we propose that like pDCs, ASDCs infiltrate

peripheral tissues during inflammation while cDC2 are present in both healthy [24] and inflamed tissues [15].

How do the 2 ASDC populations described in the manuscript correspond to the ASDCs previously identified by single-cell RNAseq? Please compare the 2 ASDC subsets to both DC2 and DC5 (PMID: 28428369) and pre-DC (PMID: 28473638). We have summarised both studies in term of phenotyping in the below table to show that DC5 and pre-DCs are the same populations. We have now added to the discussion on line 1043-1052 page 16: "The CD123^{+} and CD11c^{+} ASDC populations described in this manuscript correspond to DC5 identified by Villani et al.[4] which express AXL, Siglec-6, CD2, CX3CR1, CD33, and CD5, similar to the phenotype described for pre-DC1 and pre-DC2 (CD33^{+} CD45RA^{+} CD123^{+} CX3CR1^{+} CD2^{+} CD5^{+} Siglec-6^{+}) identified by See et al. [5]. Based on our data of **Fig 1f** showing CD11c^{+} ASDCs express lower levels of AXL and Siglec-6 than CD123^{+} ASDCs and as reported by Villani et al., we believe that pre-DC1 are the CD123^{+high} CD11c- ASDCs while pre-DC2 are the CD123$^{-/l0}$ CD11c^{+} ASDCs. Therefore, ASDCs investigated in this study are the equivalent of the previously described pre-DCs and ASDCs. Furthermore, our findings shows that ASDCs are a different population than cDC2 and that they are mature in phenotype like cDC2."

<table>
<tr><th>PMID 28428369 (CD123+ and CD11c+ ASDCs): Villani et al.</th><th>PMID 28473638 (pre-DCs): See et al</th></tr>
<tr><td>DC2: CD1C+/BDCA-1^{+}</td><td>DC2: CD45RA$^{+/-}$CD123^{-}</td></tr>
<tr>
<td>
DC5 (referred to as ASDCs): AXL+ and SIGLEC6+ and can be further subsetted on:
AXL+SIGLEC6+CD123+CD11c-
AXL+SIGLEC6+CD123+CD11c+
AXL+SIGLEC6+ CD123-CD11c+

Villani et al showed in Fig S6B (below) that both CD123+CD11C–/lo (1 pink) and <u>**the combined CD123+CD11c+ and CD123-CD11c+**</u> as CD123^{lo}CD11C+ (2 blue) in the upper panel express CD2, CX3CR1, CD33, CD5. Notably CD11c ASDCs expressed higher levels of CX3CR1, CD33 and CD5 (like DC2 or CD1C).

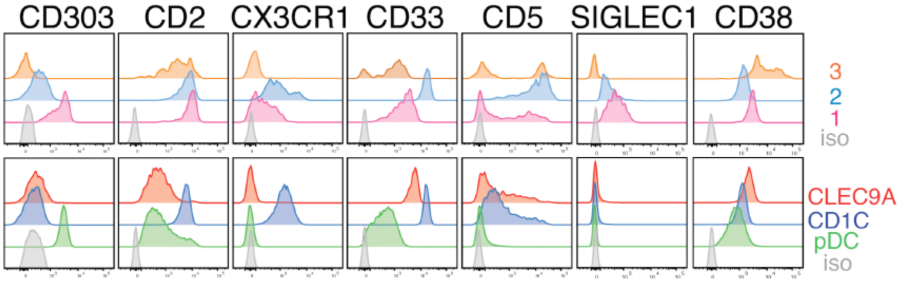

This is a similar phenotype to what was described by See et al. as pre-DCs. Therefore, pre-DCs and ASDCs are the same population.

Express co-stimulatory markers and stimulate T cell proliferation.
</td>
<td>
pre-DCs: CD33+CD45RA+CD123+CX3CR1+CD2+CD5+SIGLEC6+.
Their Fig 5F (below) showed that early pre-DCs, pre-cDC1s, pre-DC2s expressed AXL and SIGLEC6.

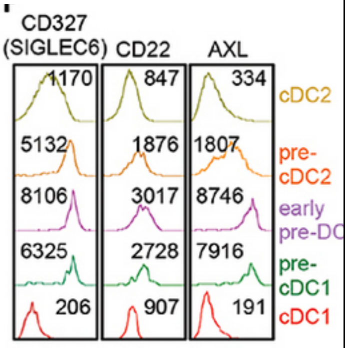

Based on our data of fig 1f (below) showing CD11c^{+} ASDCs expressing lower levels of AXL and Siglec-6 than CD123^{+} ASDCs and as reported by Villani et al., we believe that pre-DC1 are the CD123^{+high} CD11c- ASDCS while pre-DC2 are the CD123$^{-/l0}$ CD11c+ ASDCs.
</td>
</tr>
</table>

	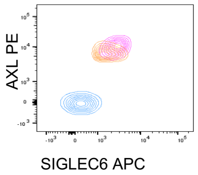<br>pre-DC express T cell costimulatory molecules and a strong capacity for naïve T cell allo-stimulation
DC6 (corresponds to pDC): purer than previously identified pDCs defined by CD123+, CD303+/BDCA-2^{+}	pDC: CD123^{+}CD33^{-}

Part III – Minor Issues: Editorial and Data Presentation Modifications
Reviewer #1:
It is not always clear in the text when ASDC purified from blood are used (versus anogenital tissue). This needs to be clarified. We have modified the manuscript to make it clear when ASDC were derived from blood versus anogenital tissue were used.

The discussion part is too long. We reduced the discussion by one page. As we needed to attend to all comments raised by the reviewer, the length is now 6 pages.

Reviewer #2:
- Please provide the gene list that was used to investigate ASDC enrichment in tissues (Figure 2b and 3). For Fig 2b, the normalised Nanostring data is provided as suppl table 1 with tab 2 showing the gene list that identified pDCs and the two subsets of ASDCs. To align the genes of fig 2b to those of Fig 3, we have now deleted from fig 3 all cytokine and chemokine gene expression as these may be expressed in a disease specific manner rather than being used to identify ASDCs and pDCs. Also to align the expression of gene in fig 2b with S6, we have amended the heat map of fig 2b from scaled expression to log2 expression of RNA counts.

-What is the status of DC2 cells in healthy and inflamed tissues? Our group has shown that DC2 exist in healthy tissues in Rhodes et al., 2021 PMID: 33846309. We have now shown in this manuscript, similar to other studies that they do also exist in inflamed tissues (Reynolds et al., 2021, PMID: 33479125). We have added to the first page of the discussion lines 1061-1062 " cDC2 are present in both healthy (24) and inflamed tissues (15)".

Please incorporate DC2 into your analyses to differentiate CD11+c ASDC from DC2. At the reviewer's request, gating in Fig 4g for the inflamed inner foreskin has been amended to show that DC2 are different from ASDCs. We have now included in Fig 4g CD1c instead of CD11b. As shown in the top third panel of 4g, there is a CD5+ CD1c- and a CD5+CDC1+ population. Further gating on these two populations in the middle third panel show i) the CD123+/intermediate SIGLEC6+ ASDCs which identifies the CD123+ CD11c- ASDC and CD123$^{-/lo}$ CD11c+ ASDCs and ii) CD123- SIGLEC6- that can be further separated into CD11c+CD14- and CD11c+CD14+. Further gating on CD11c+ CD14- identifies cDC2s as CD1c+CD163-.

-The expression of CD11c has been documented for CD123+ ASDCs (PMID: 31825848; 36866043). How does this population correlate with the CD11c+ ASDCs presented in this study? PMID: 31825848 identified the murine homolog of human AXL^{+} DCs and they refer to them as transitional DCs (tDCs) given their pDC and cDC2 characteristics. tDCs had low to high expression of CD11c. CD11c^{low} tDCs were reported to be more similar to pDCs, while CD11c^{high} tDCs correspond to cells

that were more similar to cDC2s (Figure 2A, lower left, PMID: 31825848). We believe that the CD11c^{low} tDCs are the equivalent of our CD123+ CD11c$^{-/lo}$ ASDCs which we reported to be a budding population of the right pDC island in figure 1d while the CD11c ^{high} correspond to our CD123$^{-/lo}$ CD11c+ ASDCs that were on the left island and which resemble DC2. We did not reference this study in our manuscript as it was a mouse study. In our panels of Figure 4d, we showed the CD123^{+high} CD11c- and CD123$^{-/lo}$ CD11c^{+high} ASDCs. This aligns with PMID 31825848 in that ASDCs can be further separated into **CD11c+ low** and CD11c-. In PMID: 31825848 " Isolation of DCs from Human Blood and Spleen" tDCs were gated as HLA-DR+ CD123+ BDCA1- AXL+ and then separated into CD11c- and CD11c+". We do believe that this study assessed both ASDC subsets.

Figure 2A, lower left, PMID: 31825848 Our Data

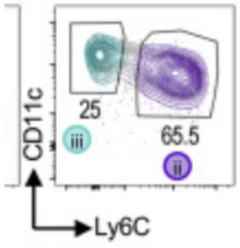

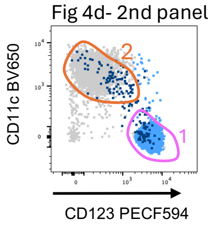

In PMID 36866043, in some experiments such as their fig 4, when Axl+ DCs were sorted into CD11c+ (66%) and CD11c- (32%) without including CD123 in their gating, both subsets of CD11c+ ASDCs and CD123^{+} ASDCs were investigated separately. However, in their figure 1 and 2, as the isolation of Axl + DCs (Axl + CD123+ CD33 int HLA-DR + Lin neg) included CD123, we believe that these experiments investigated the combined ASDCs subsets. Therefore, if CD123 cells are included in any gating used to isolate ASDCs, this will include both ASDC subsets (CD123 high and CD123-/lo CD11c+ ASDCs) if CD11c was not included. We have addressed PMID 36866043 on page 25 under the section of ASDC and HIV transmission to read on line 1744-1750: "Brouiller et al also investigated the interactions of blood derived ASDCs with HIV [31] either as combined ASDC subsets (when sorted on Axl^{+} CD123^{+}), or separated subsets (when sorted as CD11c+ and CD11c-). The high p24 detected in their CD11c^{+} Axl^{+} DCs was due to infection by HIV-1 (NL-AD8) in the presence of Vpx, thus neutralising SAMHD1. This correlates with our reasoning of high SAMHD1 expression in CD11c^{+} ASDCs which reduces their productive infection compared to CD123^{+} ASDCs expressing lower SAMHD1.".

1. RNA vs. protein expression

The authors present results from RNA sequencing data using Nanostring technology (Figure 2 and 4h), and protein expression by flow cytometry (Figure 7d) in different figures, making it challenging for readers to follow. For the ease of understanding and clarity, we have now moved Figure 4h to Fig 2c, S6 to Fig 2d, and Fig7d to 2e and therefore we have modified the text and some sections of the manuscript have been relocated to an earlier position. We have also moved figure 8 to figure 5 so the section of "Expression of HIV binding and entry receptors in Axl^{+} Siglec-6^{+} DCs and pDCs isolated from inflamed human tissues" is presented straight away after thee section "Identification of Axl^{+} Siglec^{+} DCs and plasmacytoid DCs in inflamed human tissues".

Furthermore, the data presented are not always consistent with previous results used for comparison (Figure 7d protein vs. S6 gene).

A poor correlation was shown to exist between mRNA and protein expression levels, creating concern for inferences from only mRNA expression data. Based on this, gene expression can't be at all times aligned to protein levels (PMID: 20023718 and https://doi.org/10.1038/nrg3185). Therefore, low mRNA levels can negatively correlate to high level of protein expression. Therefore, we believe the protein data in Fig 7d (now 2e) is more accurate than S6 (now 2d).

-In Fig 5. RNA (now fig 6) and Protein expression do not consistently align, as seen with the markers analyzed in Fig 5a compared to the Nanostring results in Fig2. How do the authors account these discrepancies? For the same reason mentioned above, the protein data in Fig 6a is more accurate.

-In Fig 4h (now 2c), CD123+ ASDCs exhibit the lowest RNA expression of CCR5 and CXCR4 (Villani et al.,) . How does these results compare with previous results? For the same reason mentioned above, we believe the protein data in Fig 2e is more accurate. How can the reported susceptibility to HIV-1 occur without co-receptor expression? We are confused by this statement as in our Fig 2e shows that CCR5 protein expression in all subsets was detected in all subsets at 100%. There was also higher CD4 in pDCs (>90%), followed by CD123 (>70% average) and CD11c (>60% average). Therefore, our reported susceptibility to HIV infection is mediated via CD4 and CCR5.

2. ASDCs in tissues

Apart from quantifying ASDCs in inflamed tissues using publicly available transcriptomic datasets, the authors enrolled additional patients to investigate these cells through flow cytometry and imaging mass cytometry. The results are depicted in Figure 4 and S2.

-Please provide patient information: age, sex, underlying condition, tissue collected. At the reviewer's request, we had added the patient information in supplementary tables S2 and S3.

-In Fig 4e, an FMO for Siglec-6 should be included to ensure proper gating. At the reviewer's request, we have shown an FMO for siglec6 and an FMO for AXL.

Why does the ASDC gate for this sample differ from all the others (Siglec-6 MFI > 10^3)? We have also amended Fig 4e after reanalysing the data to ensure that the ASDC gate is the same as the other specimens.

-The gating strategy in Figure 4 lacks consistency. Although different antibody panels are used in different experiments, they all include similar markers to define the 2 populations of ASDC: Axl, Siglec-6, CD123 and CD11c (Figure 1 to 4f). However, for the inner foreskin sample depicted in Figure 4g, CD11b and CD5 markers are also used.

- Please explain why a different gating strategy is used? To include a more comprehensive phenotyping and to address the reviewers query in clarifying that DC2 are a different population than CD11c ASDCs, we have now included in Fig 4g more markers as discussed above.
- Is this gating strategy similar to the one used to quantify ASDC in Figure S2? (now table S3)Yes the same gating strategy was used to identify and quantify the ASDCs in the samples listed in table S3.

-ASDCs have been previously reported in tonsils and LN, but their presence in mucosal tissues remains unknown. In addition to flow cytometry, authors utilized imaging mass cytometry to confirm the presence of ASDC in inflamed colon (Figure 4f) and discovered that ASDC are located at the periphery of a lymphoid aggregates suggestive of a tertiary lymphoid structure.

- Can the authors clarify whether they believe lymphoid aggregates are essential for the presence of ASDC presence in tissue? Very interesting questions as we are currently investigating the presences of ASDCs in lymphoid aggregates (LA). We believe that LA are not essential for the presence of ASDCs in inflamed tissues as there is a paucity of LA in labia, foreskin, and abdominal tissues. However, in intestines, LA are abundant, and this is where we located ASDCs in Fig 4f (n=1). We have added a sentence to the discussion on line 1969 to address this comment "and whether they reside in lymphoid aggregates".
- The authors write (line 528) "the tissue used in Imaging mass cytometry were from two inflamed human colons" but only 1 sample is displayed in Figure 4f. Please include the data

of the second sample or modify the material and method accordingly. We assessed three human tissues by microscopy. Initially, in the first two rectums, we used AXL and CD123 antibodies but omitted CD11c. Identifying ASDC subsets based on CD123+high AXL+ and CD123-/lo AXL+ was challenging. In the third specimen, we added CD11c as an additional marker and successfully identified pDCs and both ASDC subsets. We have now corrected the text to read one inflamed colon (line 366).

-The authors mentioned that they "did not observe pDCs or ASDCs in non-inflamed rectal tissue, confirming their presence in inflamed tissues only (Fig 4)" (line 365). However, ASDCs were also detected in non-inflamed skin tissues (Figure 4g and S2), including foreskin. Could the authors provide insight into this discrepancy? We have now made it clear that the tissues used in fig 4g and S2 were inflamed. We have amended the text on Line 430-432 to read "inflamed human tissues including abdominal skin, labia, and outer foreskin and in the epithelium and lamina propria of inner foreskin".

3. Th polarization.

I am intrigued by the little variations observed in the MLR assays illustrated in the histograms of Fig 5b and c (in the new version Fig 5 is now Fig 6). Were these experiments conducted with distinct donors or are they technical replicates? these experiments were conducted with naïve T cells and DCs/pDCs derived from 3 donors. Despite the robust proliferation observed with both types of ASDCs, the levels of IL2 quantified in the supernatant (Fig 5d now 6d) appear notably low in comparison. The reviewer raised a very good point. After analysing transcription factors, we found that Tbet was less induced in T cells cultured with the two ASDC subsets. This indicates to us that ASDCs are polarising T cells toward Th2 lineage rather than Th1 and hence there was low level of IL-2 production from T cells co-cultured with ASDCs, despite the high level of proliferation and activation in 6b and c.

4. HIV infection.

The phase termed the "1st phase of infection," involving a 2-hour exposure to HIV followed by washes and coculture with reporter T cells (Fig 7a), cannot be interpreted as indicative of different transmission modes utilized by the three cell populations tested. The histogram presented with n=3 indicates nonsignificant differences. Given that pDCs do not become infected by HIV, the observed 3.5% transmission likely represents background noise in their assay.

We agree that pDCs do not become infected. However, we are confused by this statement as our data clearly shows that pDCs do not become productively infected by HIV ie there was no second phase transfer to T cells when T cells were added 96 h post exposure of pDCs to HIV. However, we did see low levels of first phase transfer when T cells were added 2h post pDC exposure to HIV in the absence of productive infection, consistent with transfer of pDC bound virus to T cells. We have already shown in multiple manuscripts (PMID: 31227717, 25070850, 14630806) that transfer of HIV to CD4 to T cells rapidly declines with time and that after 24 hours no transfer occurs. Later (72 – 96 hours) a second phase of transfer occurred via *de novo* infection as newly formed virions bud off from the surface. This shows that using our system, later stage transfer does not occur from virions held on the cell surface or contained within intracellular compartments. Therefore, we don't believe this is background noise as this first phase transfer was consistent in three donors.

The "second phase of transmission" reveals minimal distinctions between cell types, with the difference between the two ASDC types not reaching statistical significance. This observation is consistent with the findings from the TZMBL transfer assay (Fig 7b). We have amended the results on page 16 to read "a trend of" on lines 903 and added "although this did not reach a statistical difference" on line 912. We have also amended the discussion on page 19 line 1500-1501 by adding "had a trend in transferring more HIV to CD4 T cells in first phase than $CD123^+$ ASDCs and pDCs".

The authors noted that "all characteristic genes for blood pDCs and ASDCs identified in the heat map of (Fig 2b) were downregulated upon HIV exposure" (line 231). In Figure S5 "Number of RNA counts" are displayed. How can authors rule out that variations in RNA counts result from differential susceptibility of the various DCs to cell death in their in vitro culture (18h pi)? Please note that Fig S5 is now S6. Even though all characteristic genes for blood pDCs and ASDCs were downregulated upon HIV exposure (S6a Fig), we have also detected a significant upregulation in gene expression of chemokines, IFN and Interferon stimulated genes (S6b and S6d Figs). Therefore at 18h pi, cells were viable. Can the data be normalized to housekeeping genes? We have now included in the material and method section of "Transcriptomic profile of pDCs and ASDCs via NanoString" that the data "was normalised to 15 housekeeping genes that were included in the NanoString panel. The normalisation factor for all samples ranged between 0.25 and 4.66 which is within the acceptable range of 0.1-10" (lines 2131-2133). We also added: "After the data was normalised" on line 217 in the result section, and supplementary table 1 showing in tab 1 all the normalised data from NanoString. Were the cells counted before performing Nanostring? No, but we have mock, and HIV infected an equal number of 10,000 cells.

5. The abstract.
The sentence "This indicates that the previously observed T cell stimulatory effects of pDCs are due to contaminating CD123 ASDCs" is inappropriate as this has already been proposed in previous studies. We have deleted this sentence from the abstract.
The phrase "slightly more expressed on CD11c ASDCs" is imprecise and inappropriate. The authors solely assessed expression at the RNA level by nanostring (Fig 2 b, heatmap). This should have been checked at the protein level and quantified. We have examined co-stimulatory molecules on pDCs, and the two subsets of ASDC in fig 6. The CD86 and ICAM-1 had a high level of expression (80%) across both subsets of ASDCs. However, their geometric mean fluorescence intensities (gMFI) were higher on CD11c ASDCs compared to CD123 ASDCs. We have now deleted the word slightly and amended the abstract line 34 to read "were more prevalent on CD11c" and on page 19 on line 1513-1514 to read: "Additionally, $CD11c^+$ ASDCs had a higher gMFI for CD86 and HLADR than CD123 ASDCs, indicating a more mature phenotype".

Due to the afore mentioned reasons, the authors cannot conclude that "pDCs, CD11c+ and CD123+ ASDCs expressed unique repertoires of HIV binding receptors and transmitted HIV to CD4 T cells via different modes". We have now changed the abstract line 37-43 to read: "Furthermore, we investigated the expression of chemokine receptors that facilitate ASDCs and pDCs migration from blood to inflamed tissues, their HIV binding receptors, and their interactions with HIV and CD4 T cells. For HIV infection, within 2 hours of HIV exposure, $CD11c^+$ ASDCs showed a trend in more viral transfer to T cells than $CD123^+$ ASDCs and pDCs in first phase transfer. However, for second phase transfer, $CD123^+$ ASDCs showed a trend in transferring more HIV than $CD11c^+$ ASDCs and there was no viral transfer from pDCs".

Minor points:
-Figure 5. Please verify the labelling of the different groups as discrepancies exist between the dotplots and the summative bar plots. We thank the reviewer for this comment. We have now fixed the summative bar plots.

- Figure 6. The figure legend should specify the strain of HIV-1 used in these experiments.
Figure 6 is now figure 7. We have added HIV-1 BaL to legend Fig 7 on line 2649.

- Line 203. CD83 was found to be expressed by CD11c+ ASDC in Figure 2. The text should be modified accordingly. As mentioned above, a poor correlation was shown to exist between mRNA and protein expression levels, creating concern for inferences from only mRNA expression data.

Based on this, gene expression can't be at all times aligned to protein levels (PMID: 20023718 and https://doi.org/10.1038/nrg3185). Therefore, low mRNA levels can negatively correlate to high level of protein expression and vice versa. Therefore, we believe the protein data in Fig 6a is more accurate than the RNA data of fig 2. We added on line 564-569 "It is worth noting that low mRNA levels can negatively correlate to high level of protein expression and vice versa. This was the case for CD83 and CD86. CD83 was expressed by all cells at the RNA level in Fig 2b but was not detected at the cell surface in Fig 6a. However, CD86 which had low RNA counts in all subsets was expressed at the cell surface as per the RNA counts i.e. higher gMFI on CD11c ASDCs, followed by CD123 ASDCs than pDCs ".

-In Fig 5. RNA (is now fig 6) and Protein expression do not consistently align, as seen with the markers analyzed in Fig 5a compared to the Nanostring results in Fig2. How do the authors account these discrepancies? For the same reason mentioned above, we believe the protein data in Fig 6a is more accurate.

-Line 261. Please check the reference. Thank you for prompting us that this is the wrong reference. We have deleted ref 4 and added the appropriate ref.

The following claims in conclusion may need revision as they lack experimental evidence support:
- Line 366. "Thus, like pDCs, ASDCs infiltrate peripheral tissues during inflammation." We amended this to read on line 1059-1060 "Thus, we propose that like pDCs, ASDCs infiltrate peripheral tissues during inflammation".

-Line 357. The statement should be slightly modified. See PMID 36866043.
Thank you for bringing this to our attention. Line 357 (now 1743-1749) indicated that Brouiller et al investigated only the combination of both CD123^{+high} and CD123$^{-/lo}$ CD11c+ASDCs interactions with HIV. As we were asked to shorten the discussion, this line has been corrected under the section of ASDC and HIV transmission to read that depending on their sorting strategy Brouiller et al have looked at ASDCs interactions with HIV either as the combined ASDC subsets together or as two separate subsets of ASDCs: "Brouiller et al also investigated the interactions of blood derived ASDCs with HIV [31] either as combined ASDC subsets (when sorted on Axl^{+} CD123^{+}), or separated subsets (when sorted as CD11c+ and CD11c-). The high p24 detected in their CD11c^{+} Axl^{+} DCs was due to infection by HIV-1 (NL-AD8) in the presence of Vpx, thus neutralising SAMHD1. This correlates with our reasoning of high SAMHD1 expression in CD11c^{+} ASDCs which reduces their productive infection compared to CD123^{+} ASDCs expressing lower SAMHD1."

- Line 430. "Furthermore, we found that CD11c+ ASDCs expressed higher levels of the HIV restriction factor SAMHD1 than CD123+ ASDCs, explaining their lower levels of infection and reduced capacity to mediate late/second phase transfer." The reduced susceptibility to HIV-1 infection of CD11c+ cells was only indirectly demonstrated by measuring infectious virions in culture supernatant and has not been directly proven. Yes we agree that we have not proven directly that CD11c showed a reduced infection compared to CD123 ASDCs. HIV infection of CD11c+ASDCs and CD123+ASDCs was performed on 30,000-60,000 cells as per our material and methods. We tried to assess infectivity of both ASDC subsets via intracellular p24 expression, but this was not feasible to perform (despite multiple attempts) as with all the steps needed to stain for flow cytometry, we ended up with too few live cells to gate on to measure direct infection. Therefore, we assessed the infection state of ASDCs by 1) inoculating TZMBL1, a CD4 T cell line, with the supernatants collected from infected cells at 96 h post infection and 2) via JLTR infection (fluoresce green) when co-cultured with HIV infected ASDCs for 96h. we have now added on page 19 line 1506-1509 "We attempted to directly assess the productive infection of CD123+ ASDCs and CD11c+ ASDCs via p24 expression. However, due to low numbers of blood derived ASDCs, we had insufficient live cells for accurate measurement". We have also deleted from the result section of "HIV viral transfer from

blood-derived Axl^+ $Siglec\text{-}6^+$ DCs and plasmacytoid DCs to CD4 T cells" the justification of HIV viral transfer from all DC subsets as this is a duplication of what is in the discussion section.

Figure Files:
While revising your submission, please upload your figure files to the Preflight Analysis and Conversion Engine (PACE) digital diagnostic tool, https://pacev2.apexcovantage.com. PACE helps ensure that figures meet PLOS requirements. To use PACE, you must first register as a user. Then, login and navigate to the UPLOAD tab, where you will find detailed instructions on how to use the tool. If you encounter any issues or have any questions when using PACE, please email us at figures@plos.org.
Data Requirements:
Please note that, as a condition of publication, PLOS' data policy requires that you make available all data used to draw the conclusions outlined in your manuscript. Data must be deposited in an appropriate repository, included within the body of the manuscript, or uploaded as supporting information. This includes all numerical values that were used to generate graphs, histograms etc.. For an example see here on PLOS Biology: http://www.plosbiology.org/article/info:doi/10.1371/journal.pbio.1001908#s5.
Reproducibility:
To enhance the reproducibility of your results, we recommend that you deposit your laboratory protocols in protocols.io, where a protocol can be assigned its own identifier (DOI) such that it can be cited independently in the future. Additionally, PLOS ONE offers an option to publish peer-reviewed clinical study protocols. Read more information on sharing protocols at https://plos.org/protocols?utm_medium=editorial-email&utm_source=authorletters&utm_campaign=protocols
