# Supplementary material for: Characterising plasmacytoid and myeloid AXL+ SIGLEC-6+ dendritic cell functions and their interactions with HIV
Source: PLoS Pathog. 2024 Jun 26;20(6):e1012351. doi: 10.1371/journal.ppat.1012351 (PMC11233022; doi:10.1371/journal.ppat.1012351)
Supplement: Attachment — Submitted filename: Response #2 to the reviewer.docx [file ppat.1012351.s013.docx]

Thank you for all the comments. Below are our responses to each comment in blue font and what have been included in the manuscript is in red font. Line numbers refer to the tracked changes version of the manuscript.

Reviewer #2: Page 4. On the comparison of CD123+ and CD11c+ ASDC gene signature with previous RNAseq data sets. The authors comprehensively and thoroughly compared the phenotype of ASDC populations with previous articles. However, it is also important to validate the gene signature obtained for the 2 ASDC populations described with known data sets. Especially as the gene signature the authors used (Fig 2b, Table S1) are issued from ex vivo DC after culture (Mock- or HIV-culture), which could impact greatly gene expression.

To address the above query, we have amended the text on page 7 line 131 to read:

Since our NanoString panel was targeting genes involved in immunology, we were limited in identifying all genes that define pDCs, ASDCs and all those that discriminate between CD123+ and CD11c+ ASDCs as reported by Villani et al. After normalizing the NanoString data, *TLR7* was detected in pDCs only; *IRF8, IRF7, APP, TCF4* and *LILRA4* were expressed more highly in pDCs and CD123+ ASDCs; *CD5* was exclusively identified in ASDCs. Out of all genes that discriminated between CD123+ and CD11c+ ASDCs, our NanoString panel showed *LILR4* expression only in CD123+ ASDCs. As all the above genes show a similar pattern to that reported by Villani et al in differentiating ASDCs and pDCs, this implies that the gene signature for ASDCs did not change upon 18 h of culture prior to NanoString testing. We also noted pDCs were CD45RA+, CD11c+ ASDCs were CD45RO+ while CD123 ASDCs were a mix of CD45RO+ and RA+. A higher expression of the costimulatory molecule *CD86* was detected in CD11c+ ASDCs compared to CD123+ ASDCs. This also correlated with its higher protein expression on CD11c+ ASDCs as previously reported (Villani et al). Interestingly, we also identified *indoleamine 2,3-dioxygenase (IDO), CLEC5A* and *LAMP-3* as being highly expressed by CD11c^+^ ASDC, while CLEC4A, *FCER1G* and *FCGR2B* were more expressed in CD123+ ASDCs. Furthermore, our data showed that *ICAM-1* and *HLA-DR* were expressed by pDCs and ASDCs.

About SAMHD1:

DCs are thought to express high levels of SAMHD1, which render them resistant to HIV-1 infection. Previous reports also show that addition of Vpx to HIV-1 particles that induces SAMHD1 degradation leads to 10-time increase of Axl+Siglec6+ DC infection rate (PMID: 31591213). In Figure 2e, the authors report a low expression of SAMHD1 protein (2-7%). I acknowledge the difficulty to perform intracellular staining with very few cells. However, as the authors emphasize a differential susceptibility to HIV-1 infection of the 2 ASDC populations and relate it to SAMHD-1 expression, it would be important to show positive control of SAMHD1 staining (e.g. on THP1 cells) and validate the results maybe by pooling different donors.

We thank the reviewer for their observations of SAMHD1 expression. The paper cited, PMID: 31591213, investigated SAMHD1 expression by mRNA levels whereas we examined SAMHD1 by protein expression. As RNA and protein expression levels of a single marker can differ, it is not possible to accurately correlate these two results. However, we have re-analysed our SAMHD1 protein expression data in figure 2e and adjusted the gating to be tighter with the FMO control (a representative plot is included below). Both ASDC subsets and pDCs now demonstrate higher levels of SAMHD1 expression.

To address the reviewers suggestion of including a positive control for SAMHD1, several studies have previously shown MDDCs to express high levels of SAMHD1 protein expression (PMID: [25926647](https://pubmed.ncbi.nlm.nih.gov/25926647), PMID: [23269793](https://pubmed.ncbi.nlm.nih.gov/23269793)). We have subsequently stained MDDCs with SAMHD1 using the same antibody and staining protocol used for our data generated previously. Our MDDC staining shows 90% SAMHD1 expression (shown below), confirming the antibody’s effectiveness. Alongside the MDDC staining, we repeated SAMHD1 staining on ASDCs and pDCs with an additional donor which yielded similar results to the previous 4 donors.

We have updated figure 2e to reflect the new analysis of our data and to include the fifth donor.

SAMHD1 APC

HLA-DR BUV395

**pDC**

**CD11c+ ASDC**

**CD123+ ASDC**

39.9%

37.4%

25.9%

FMO

SAMHD1+

SAMHD1 APC

FSC-A

90%

MDDC FMO

MDDC SAMHD1+

About cDC2. Page 3.

Figure 6A from PMID 28428369 represent peripheral DCs at steady state. To exclude any overlap between cDC2 and ASDC, it is important to show that ASDCs do not express CD1c. The gating strategy used in Figures 1, 4 and S5 do not allow to verify this particular point of importance.

We thank the reviewer for taking their time to ensure our results have validity, however, previous literature demonstrates that CD11c+ ASDCs do express CD1c and hence CD1c is not sufficient to discriminate between ASDCs and cDC2. In figure 6A of Villani et al (PMID 28428369), ASDCs are identified as Axl^+^ Siglec-6^+^ and cDC2 as Axl^-^ Siglec-6^-^ CD123^-^ CD11c^+^ CD1c^+^. The ASDCs are gated upstream of cDC2s which does not preclude ASDCs from expressing CD1c. Furthermore, Figure S5C-D of Villani et al indeed demonstrates CD1c expression on the CD11c^+^ CD123^lo^ ASDC subset. These findings were also verified in the work of See et al (PMID 28473638) who showed CD123^lo^ CD11c^hi^ pre-DCs express CD1c in figure 6B.

Our gating is similar to Villani et al in that ASDCs are identified upstream of cDC2s and separated by their unique expression of Axl, Siglec-6 or CD123. We have included a figure below from our blood and tissue data to demonstrate that we also see CD1c expression on CD11c+ ASDCs but not on CD123+ ASDCs, in accordance with the results of Villani et al and See et al.

CD1c BV650

Siglec-6 BV480

**Blood**

**Tissue**

**(inner foreskin**

**lamina propria)**

**Villani et al Fig S5**

**See et al Fig 6**

D

C

**CD11c+ ASDC**

**CD123+ ASDC**

**cDC2**

Ruffin et al (PMID 31591213) present ASDCs to be CD1c-. However, this gating was performed on only the CD123^hi^ ASDC population, which we, as well as Villani et al and See et al, showed do not express CD1c. It is only the CD11c+ ASDC population which expresses CD1c.

To further emphasise the distinctiveness of ASDCs compared to cDC2s and other DCs we performed a tSNE clustering on 3 of our tissue samples: labia epidermis, labia dermis and abdomen epidermis. The clustering clearly demonstrates both pDCs and ASDCs cluster separately to Langerhans cells and CD1c^hi^ DCs.

**Low**

**High**

Labia Epidermis

Labia Dermis

Abdomen Epidermis

ASDC

pDC

Langerhans cells

CD1c hi DC

Based on these above points we do not feel the gating strategy used in Figures 1, 4 and S5 need to be altered.
